# Supplementary material for: The SGLT2 inhibitor canagliflozin suppresses growth and enhances prostate cancer response to radiotherapy
Source: Commun Biol. 2023 Sep 8;6:919. doi: 10.1038/s42003-023-05289-w (PMC10491589; doi:10.1038/s42003-023-05289-w)
Supplement: Supplementary file 2 — Supplementary Figures and Tables [file 42003_2023_5289_MOESM2_ESM.pdf]

Supplementary figures

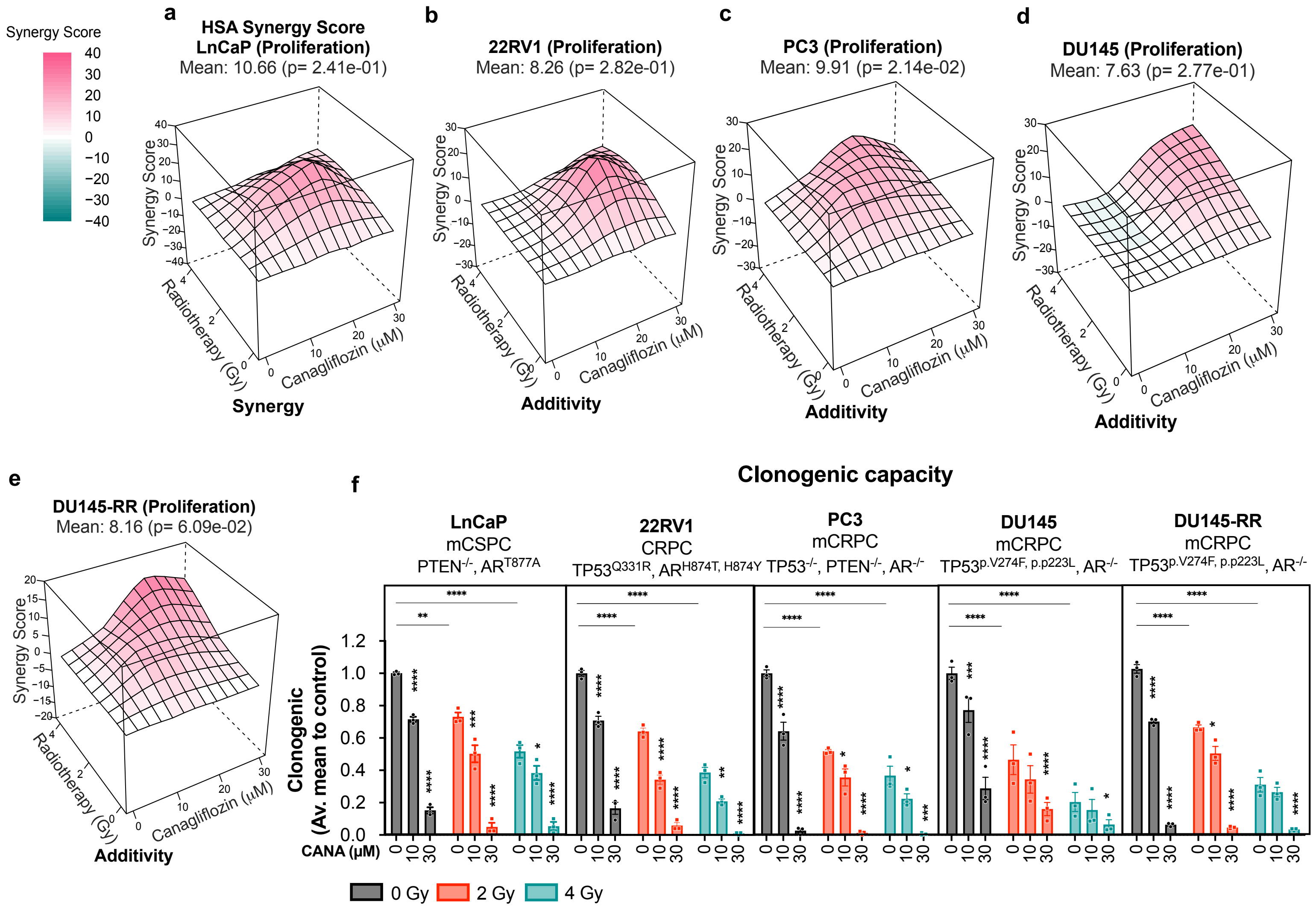

g

Cell Clonogenic-Canaliflozin IC50				
Radiation (Gy)	PC3	22RV1	LnCap	DU145
0	15.01μM	16.6264μM	16.90μM	20.81μM
2	15.40μM	11.8672μM	14.90μM	24.7799μM
4	16.95μM	11.232μM	15.20μM	21.03μM
	Canagliflozin (μM)			

Cell Proliferation-Canaliflozin IC50				
Radiation (Gy)	PC3	22RV1	LnCap	DU145
0	19.9234μM	21.2743μM	20.44μM	40.85μM
2	20.8702μM	20.7639μM	20.67μM	42.5μM
4	21.93μM	21.98μM	22.2296μM	47.13μM
	Canagliflozin (μM)			

Supplementary Figure 1. Effects of canagliflozin (CANA) or radiotherapy (RT) on the cell proliferation and Clonogenic survival. **(a-e)** The synergy score for cellular proliferation induced by the combined treatment of CANA and RT, as well as their individual effects in prostate cancer cell lines (PrCa cells). Highest Single Agent (HSA) synergy score of four PrCa cell lines with different mutation profiles (LnCaP, 22RV1, PC3, DU145) and one radio-resistant cell line (DU145-RR) analyzed on proliferation assay. HSA mean score of (10 or higher indicates synergism, a score between (+10 and -10) indicates additivity, and a score =< -10 indicates antagonism). **(f)** Clonogenic assay performed with prostate cancer (PrCa) cell lines treated with single agent or combined treatments, canagliflozin 0-30μM and radiation (RT) (0-4Gy), n=3. **(g)** Canagliflozin IC50 values table for both individually and in combination with radiation doses ranging from 0 to 8Gy, for clonogenic and proliferation assays conducted on prostate cancer cell lines. Two-way analysis of variance (ANOVA) with the post hoc Tukey's multiple comparisons test was used to evaluate differences between the treatment and control groups. The results were obtained by at least three separate experiments n=3. The data shown is mean ± SEM.

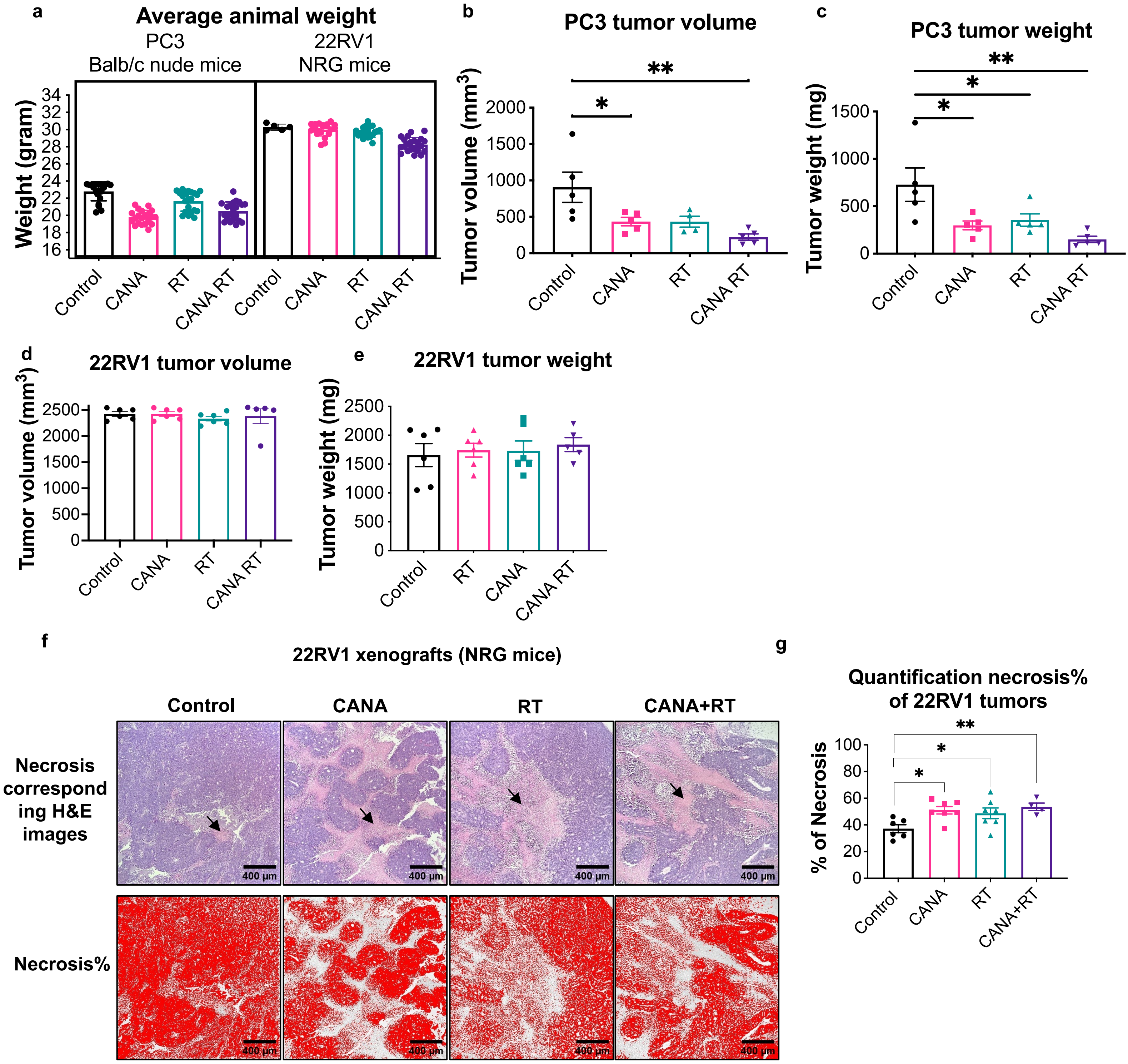

Supplementary Figure 2. Assessing the impact of Canagliflozin (CANA) and Radiotherapy (RT) Combination *in vivo*. (a) Average animal weight, (**b-e**) Ex vivo tumor weight (mg) and ex vivo tumor volume (mm^3), (**b-c**) in PC3 xenograft nude mice, and (**d-e**) 22RV1 xenografts NRG mice, both were measured at the endpoint. **(f-g)** Representative images of IHC examination of the necrosis and its quantification of tumors from 22RV1 NRG mice. **(f)** Tumors stained with H&E from 22RV1 NRG mice in the control, canagliflozin, radiation, and combination treatment groups. In the control and treatment groups, arrows on the images point to the necrotic area. Necrosis ratio images were created using ImageJ software. The red colour represents the non-necrotic vital areas, while the white colour represents the necrotic areas. **(g)** Quantification of necrosis ratio (%) images. To quantify the necrosis % in the 22RV1 xenograft tumor, all slides were stained with H&E and the whole section was quantified using ImageJ software, following the ImageJ user guide for tissue quantification found on the NIH ImageJ website (https://imagej.nih.gov/ij/index.html). We calculated necrotic area as follows: Necrotic Area = Total Area – Viable Tissue Area, and Necrotic Tissue Percentage = Necrotic Area / Total Area. Ordinary one-way anova, Tukey's multiple comparison, n=6/group were used for statistical analysis, asterisks represent of p value, * = $p<0.05$, and ** = $p<0.01$. The data shown is Mean ± SEM.

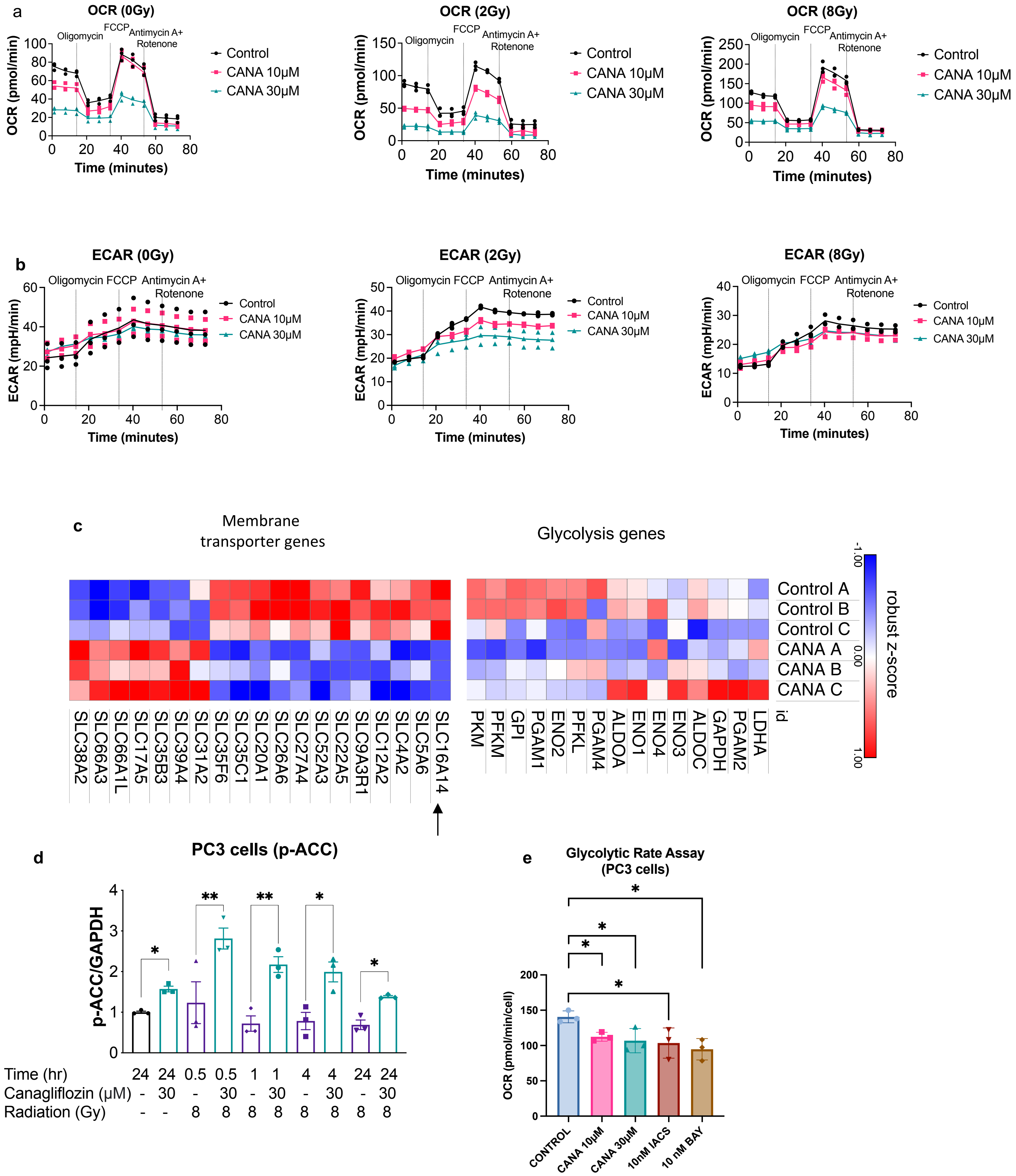

Supplementary Figure 3. (a-b) Effects of canagliflozin and radiation (RT) treatments on (**a**) oxygen consumption rate (OCR), and (**b**) extracellular acidification rate (ECAR), raw data. **(c)** Regulation of expression of glycolytic related genes of the **"**glycolysis**"** Gene Ontology (GO) term and membrane transporter genes. All glycolysis related genes are not significantly regulated by canagliflozin, FDR q-value > 0.05. All membrane transporter genes are significantly downregulated by canagliflozin, FDR q-value < 0.05. (**d**) Immunoblotting assay quantification for phosphorylated-ACC(Ser^{79})/GAPDH. (e) Normalized basal OCR values from the glycolytic rate assay (GRA) experiment in PC3 cells treated with canagliflozin, IACS-010759, or BAY-872243. Cell number normalization was performed using crystal violet for the cells. The significance of the time course experiment at each treatment dosage was determined using a two-way analysis of variance (ANOVA), asterisks represent of p value, * = p<0.05, and ** = p<0.01. The data shown is mean ± SEM; n=3. Ordinary one-way ANOVA with the post hoc Tukey's multiple comparisons test was performed for figure e, to determine if there were significant changes between the treatment and control groups.

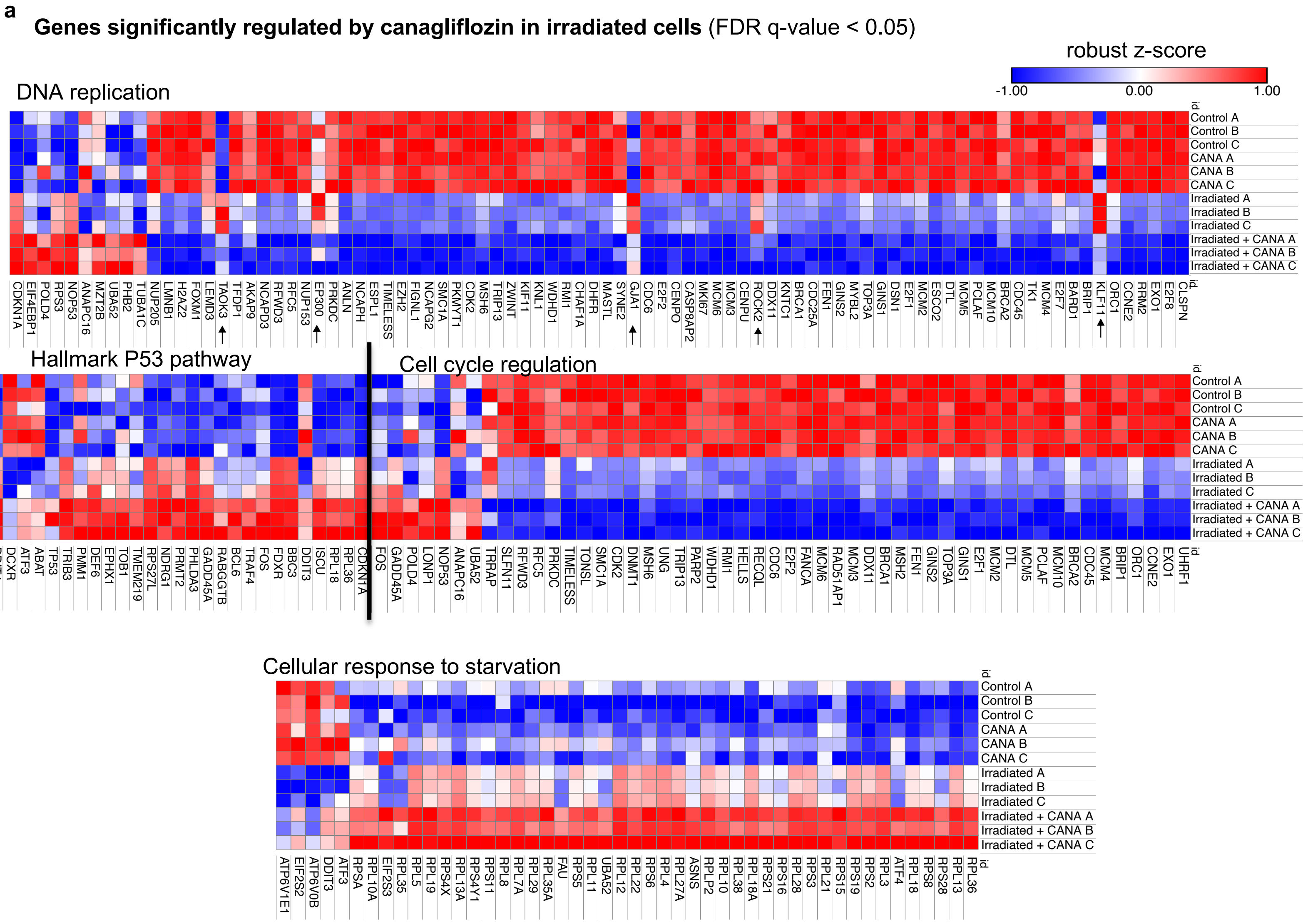

Supplementary Figure 4. Regulation of expression of genes genes involved in "cell cycle, DNA replication, p53 pathway and cellular response to starvation" Gene Ontology (GO) term. (**a**) Heatmap diagrams illustrating effects of canagliflozin (10μM) (CANA), radiation (5Gy) (RT) or combined treatment in 22RV1 cells were generated from RNAseq analysis data (False Discovery Rate (FDR) p-value < 0.05). Normalized log2 feature counts are used to represent gene expression (with robust z-score). The blue to red scale represents downregulated to upregulated from -1 to 1.

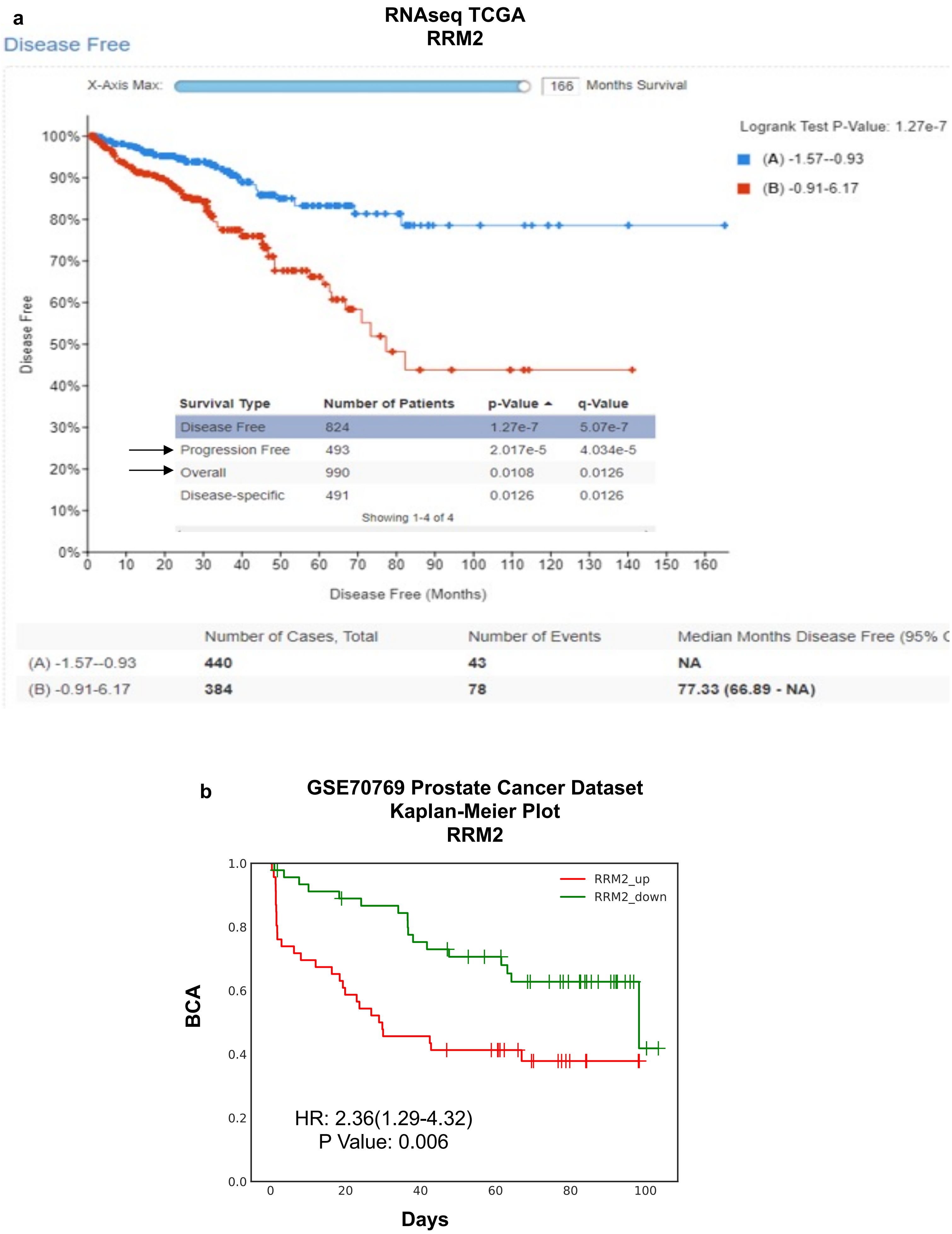

Supplementary Figure 5. Kaplan-Meier plots indictaing the potential prognostic value of *RRM2* gene. The cBioPortal for cancer genomics (http://www.cbioportal.org) and the Prostate Cancer Transcriptome Atlas (PCTA) (http://www.thepcta.org) engines were used to analyze data. (**a**) Kaplan-Meier plot of The Cancer Genome Atlas (TCGA) data analyzed (cBioPortal). Graph illustrates relationship with Disease-Free survival, table insert describes also the relationship with progression-free, disease-Specific and overall survival. (**b**) Analysis of Cancer Research UK Cambridge Institute cohort (GSE70769) data (PCTA). Kaplan-Meier plot of biochemical recurrence-free survival (BCA).

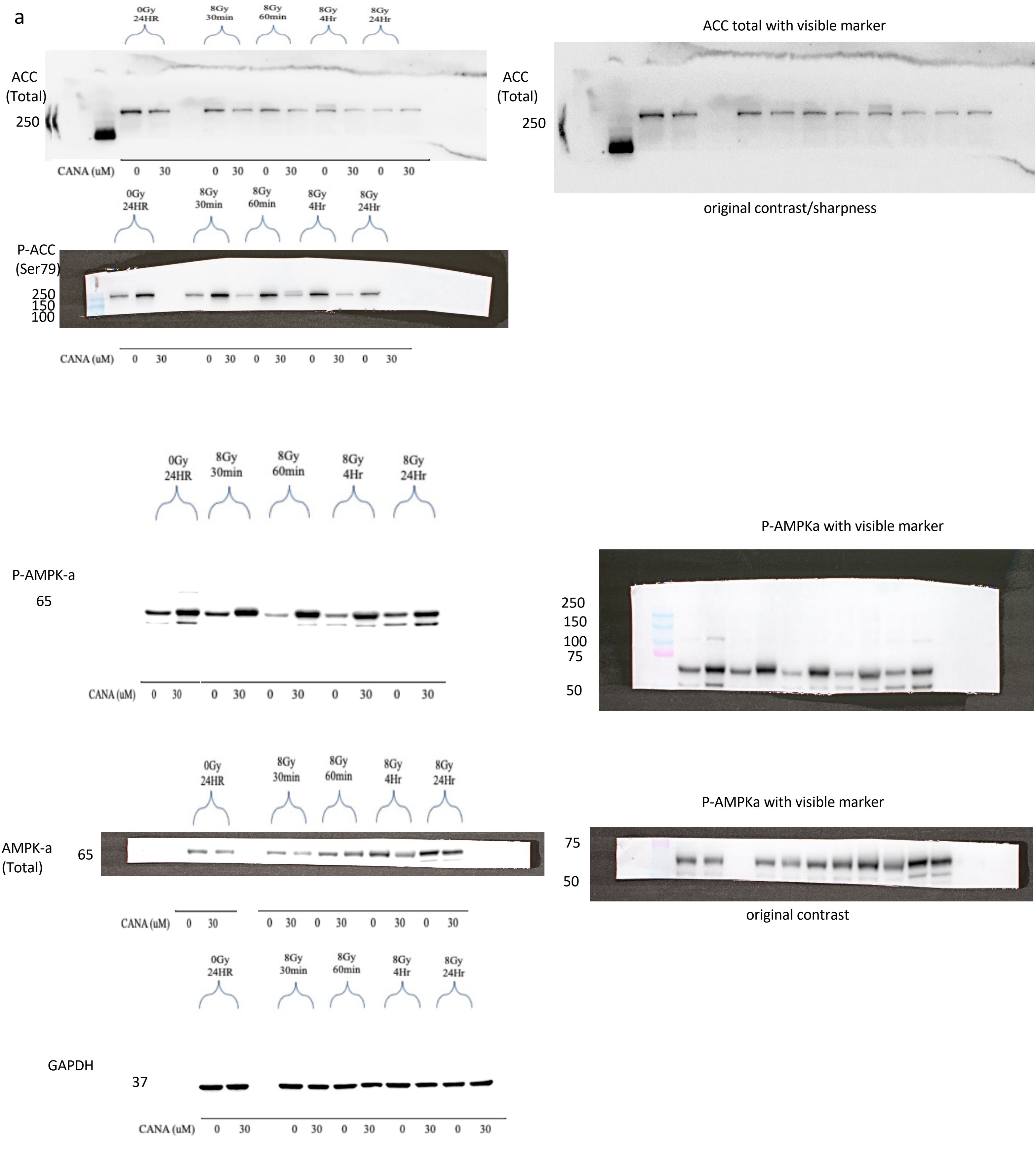

Supplementary Figure 6. Supplementary blots a: The supplementary blots a are for Figure 4h (left side) contain the blots for ACC (total and phosphorylated-ACC ser79), AMPK-alpha (total and phosphorylated AMPK-alpha at Thr172), and a representative GAPDH control blot. The blot on the right side shows the visible molecular weight ladder, original contrast and/or sharpness. The p-AMPK-alpha band in the manuscript has been spaced to match the other blots between CANA 30 and 8Gy 30min. Figures may have a different aspect ratio than the image in the manuscript due to the size of the blot, as we are showing the fully uncropped and unedited images here.

b

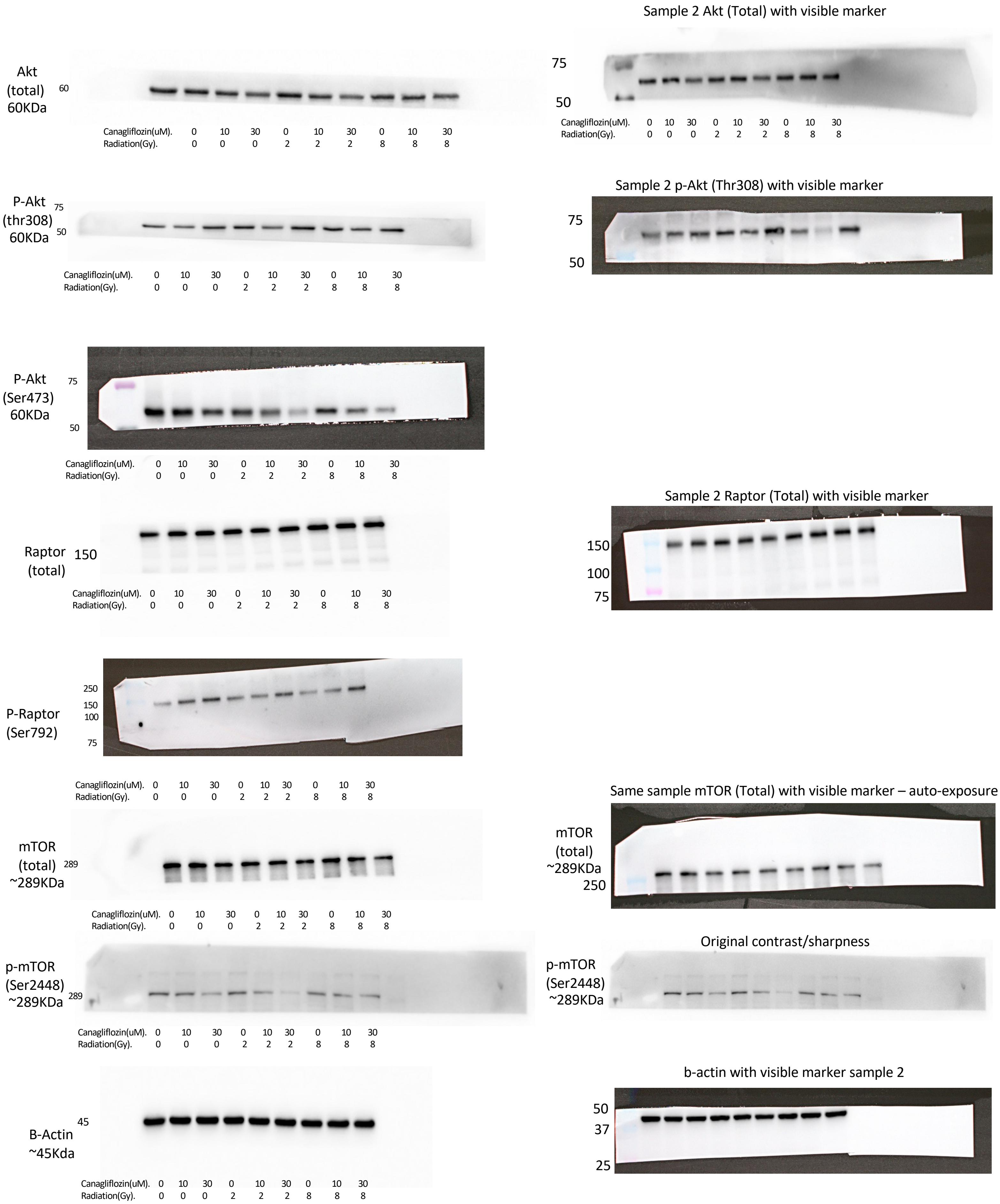

Sample 2 Akt (Total) with visible marker
Akt (total) 60KDa
60
75
50
Canagliflozin(uM). 0 10 30 0 10 30 0 10 30
Radiation(Gy). 0 0 0 2 2 2 8 8 8
Sample 2 p-Akt (Thr308) with visible marker
P-Akt (thr308) 60KDa
75
50
P-Akt (Ser473) 60KDa
75
50
Sample 2 Raptor (Total) with visible marker
Raptor (total)
150
100
75
P-Raptor (Ser792)
250
150
100
75
Same sample mTOR (Total) with visible marker – auto-exposure
mTOR (total) ~289KDa
289
250
Original contrast/sharpness
p-mTOR (Ser2448) ~289KDa
289
b-actin with visible marker sample 2
B-Actin ~45Kda
45
50
37
25

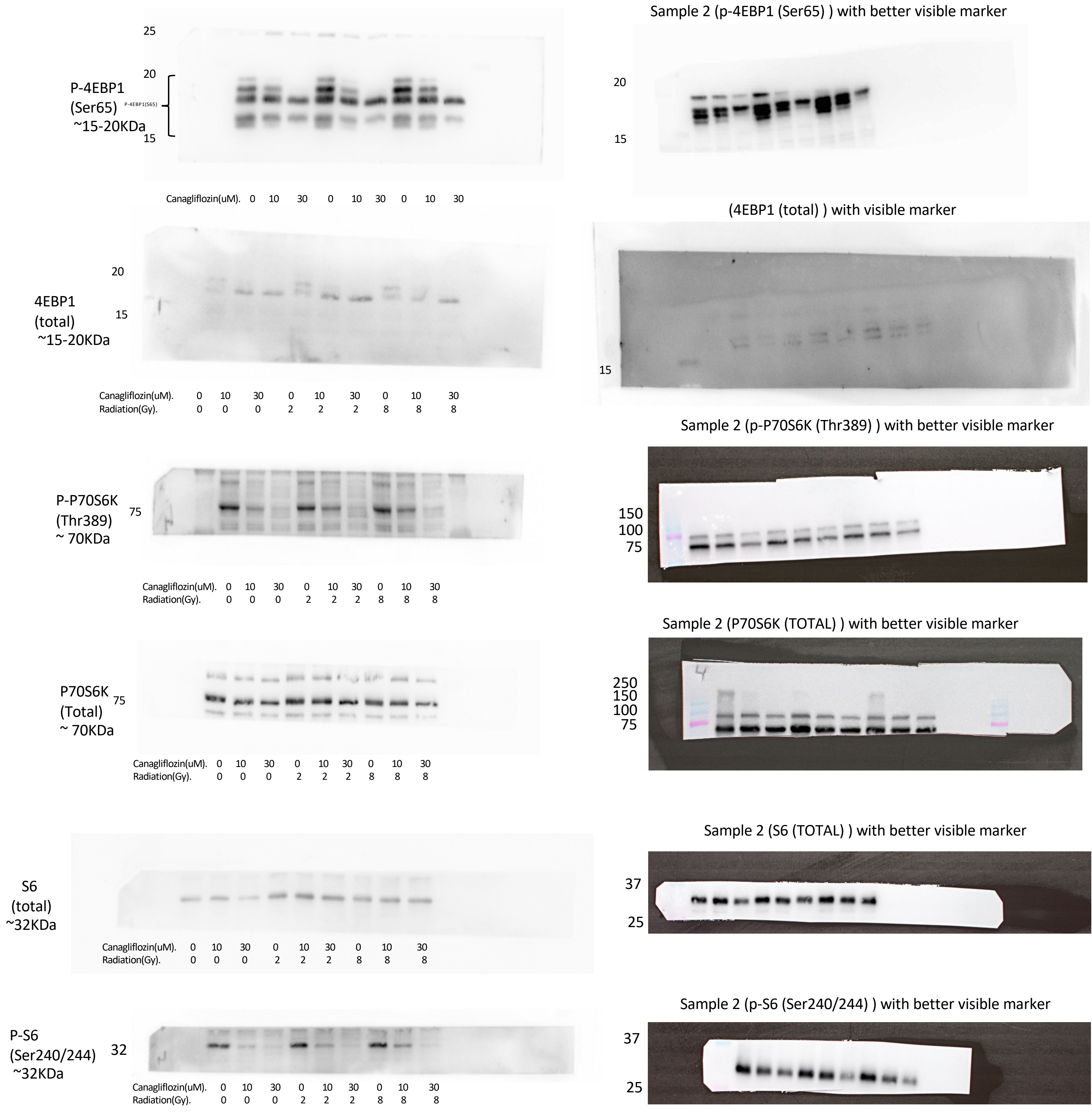

Supplementary Figure 6. Supplementary blots b: The supplementary blots b contain the original blots for Figure 5a (mTOR Pathway), including AKT (total and phosphorylation at thr308 and Ser473), Raptor (total and phosphorylation at Ser792), mTOR (total and phosphorylation at Ser2448), 4-EPBP1 (total and phosphorylation at Ser65), P70S6K (total and phosphorylation at Thr389), S6 (total and phosphorylation at Ser240/244), and a representative β-actin control blot. The blots on the right side shows the visible molecular weight ladder. Figures may have a different aspect ratio than the image in the manuscript due to the size of the blot, as we are showing the fully uncropped and unedited images here.

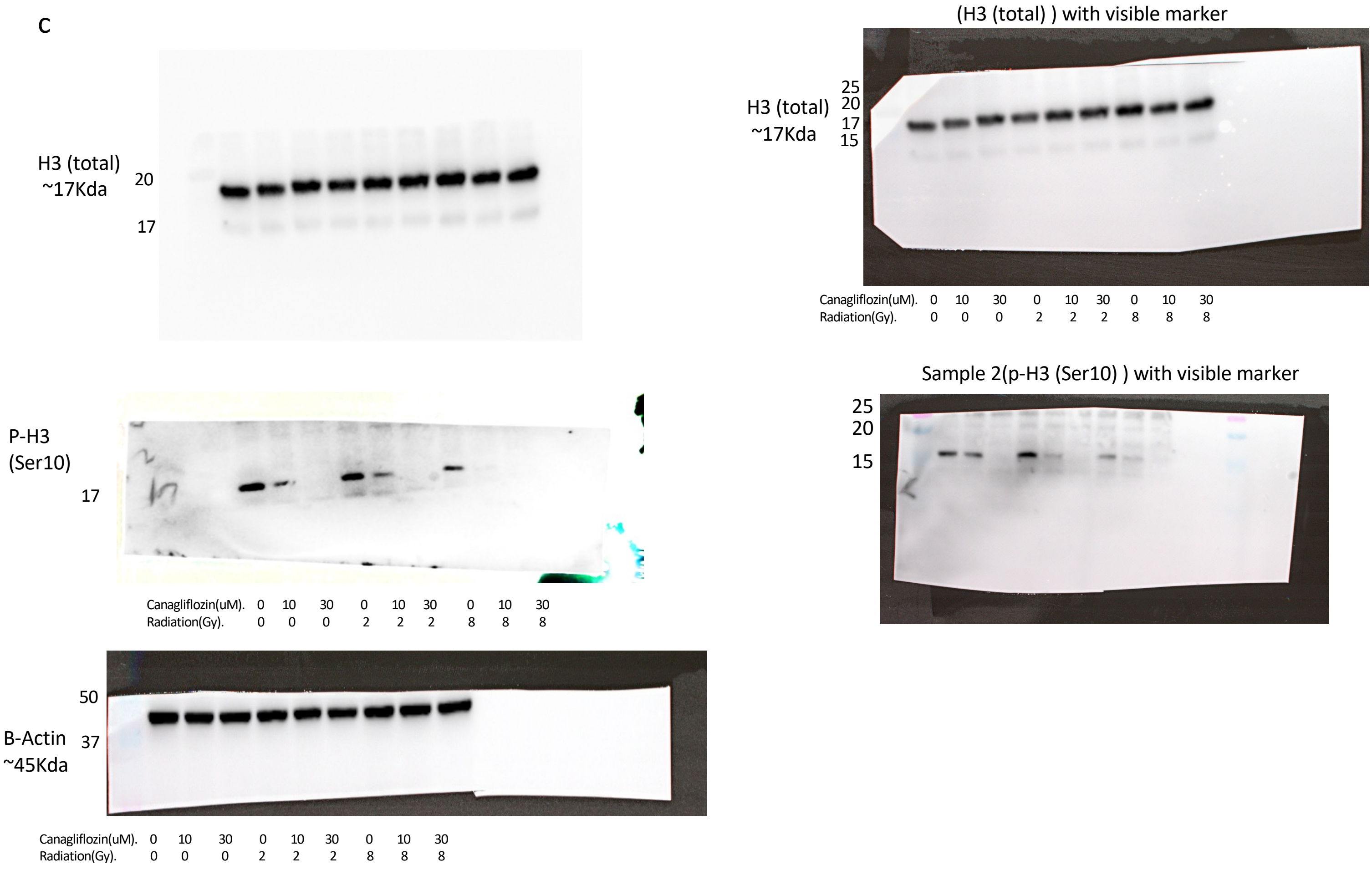

Supplementary Figure 6. Supplementary blots c: The supplementary blots c contain the original blots for Figure 5a (DNA replication), including H3 (total and phosphorylated H3 at ser10), and a representative β-actin control blot. The blots on the right side shows the visible molecular weight ladder. Figures may have a different aspect ratio than the image in the manuscript due to the size of the blot, as we are showing the fully uncropped and unedited images here.

d

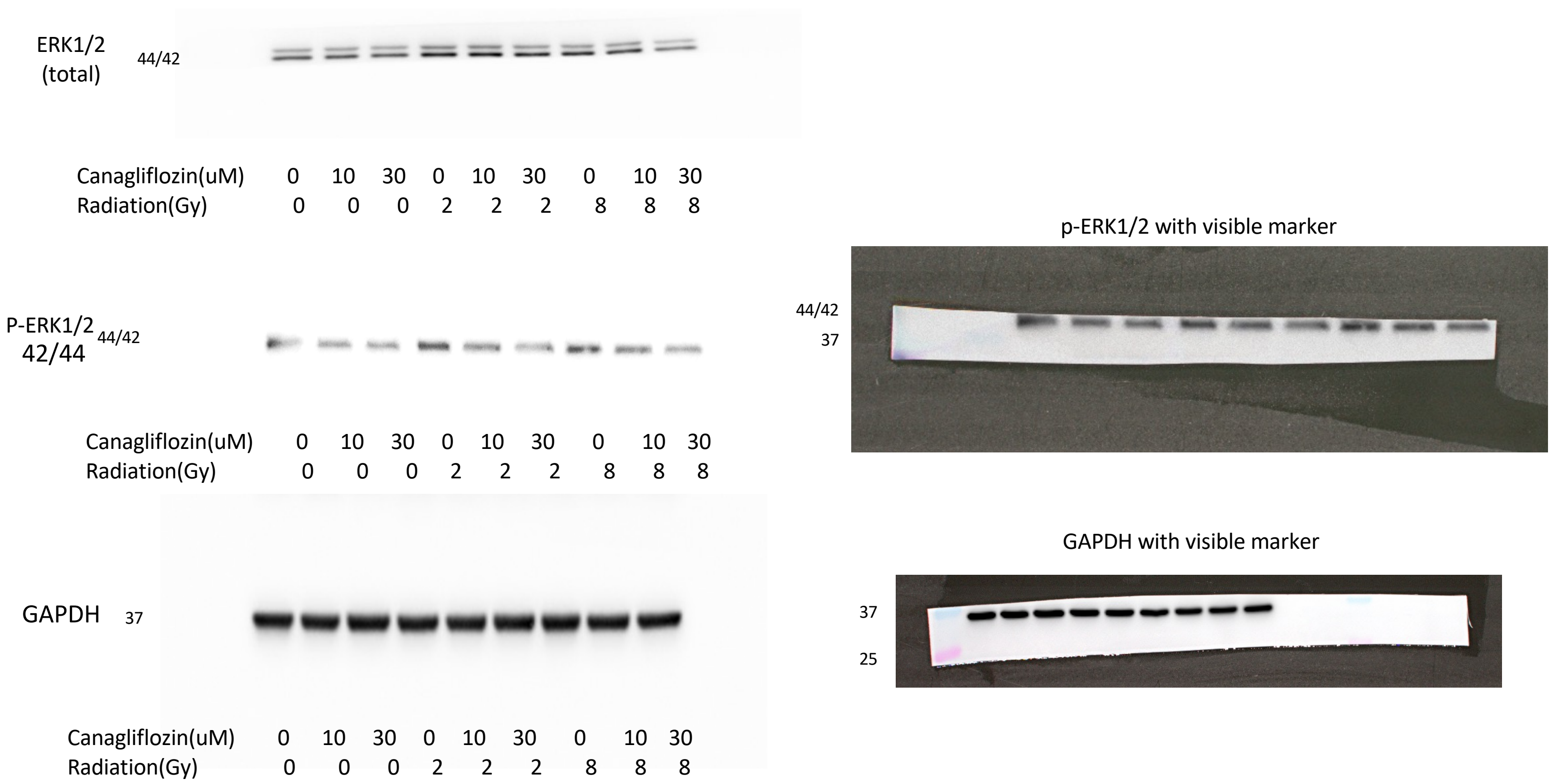

Supplementary Figure 6. Supplementary blots d: The supplementary blots 4 contain the original blots for Figure 5a (Erk/MAPK Pathway), including ERK (total and phosphorylated Erk1/2 at 42/44), and a representative GAPDH control blot. The blots on the right side shows the visible molecular weight ladder. Figures may have a different aspect ratio than the image in the manuscript due to the size of the blot, as we are showing the fully uncropped and unedited images here.

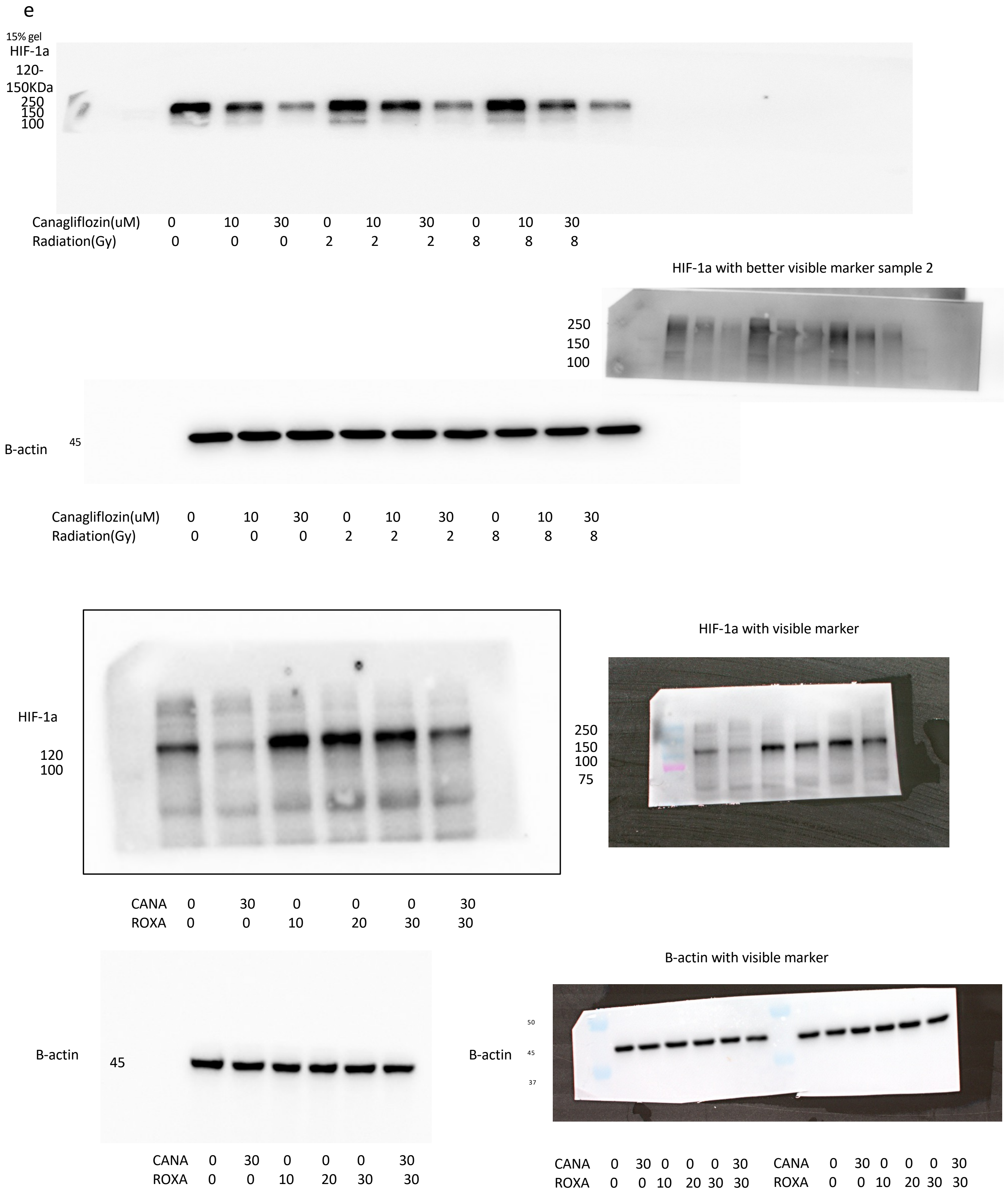

Supplementary Figure 6. Supplementary blots e: The supplementary blots 5 contain the original blots for Figure 6a and Figure 6c (Hif-1aplha), and the representative b-actin control blot. The blots on the right side shows the visible molecular weight ladder. Figures may have a different aspect ratio than the image in the manuscript due to the size of the blot, as we are showing the full image.

f

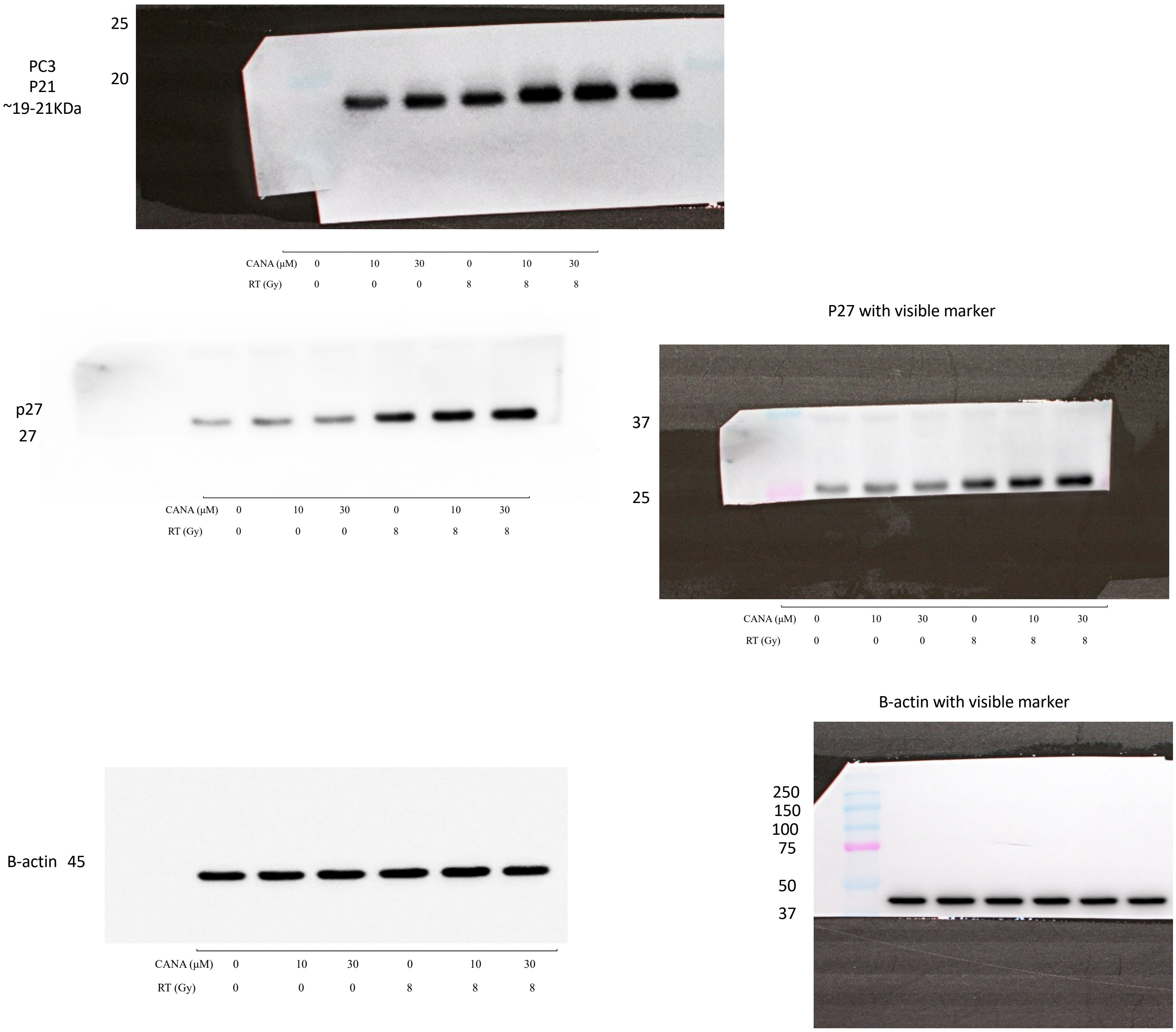

Supplementary Figure 6. Supplementary blots f: The supplementary blots 6 contain the original blots for Figure 7a for PC3 cells cell cycle checkpoints (P21 and P27), and the representative B-actin control blot. The blots on the right side shows the visible molecular weight ladder. Figures may have a different aspect ratio than the image in the manuscript due to the size of the blot, as we are showing the full image.

g

22rv1-p21
~19-21KDa

20

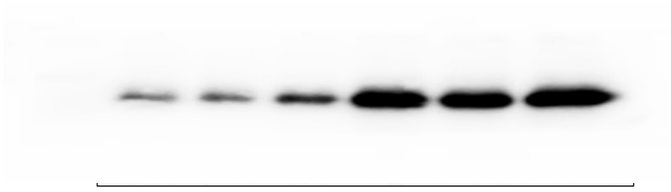

CANA (μM)	0	10	30	0	10	30
RT (Gy)	0	0	0	8	8	8

P21 with visible marker

20

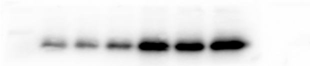

Another P21 sample with visible marker

25
20

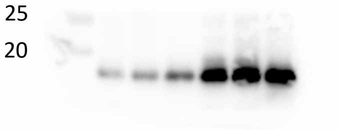

22rv1-b-actin

45

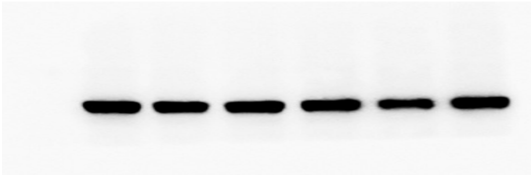

CANA (μM)	0	10	30	0	10	30
RT (Gy)	0	0	0	8	8	8

22RV1 cells
P-P53 (Ser15)

75
50

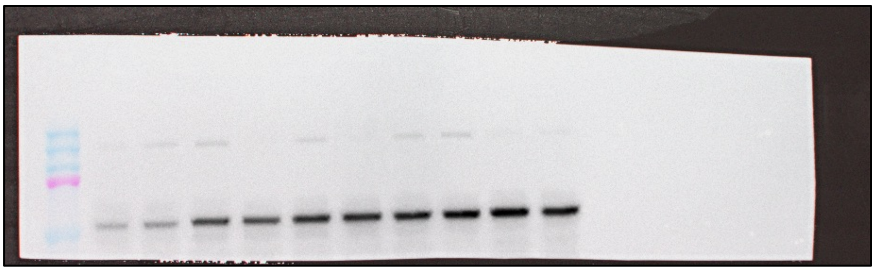

GAPDH
37

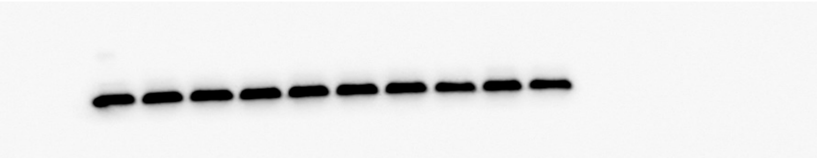

Time post-RT (hr)	24	24	0.5	0.5	1	1	4	4	24	24
CANA (μM)	0	30	0	30	0	30	0	30	0	30

GAPDH sample 2 with visible marker

37
25
20

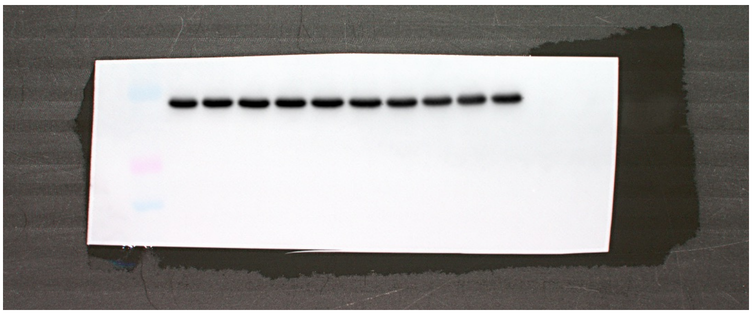

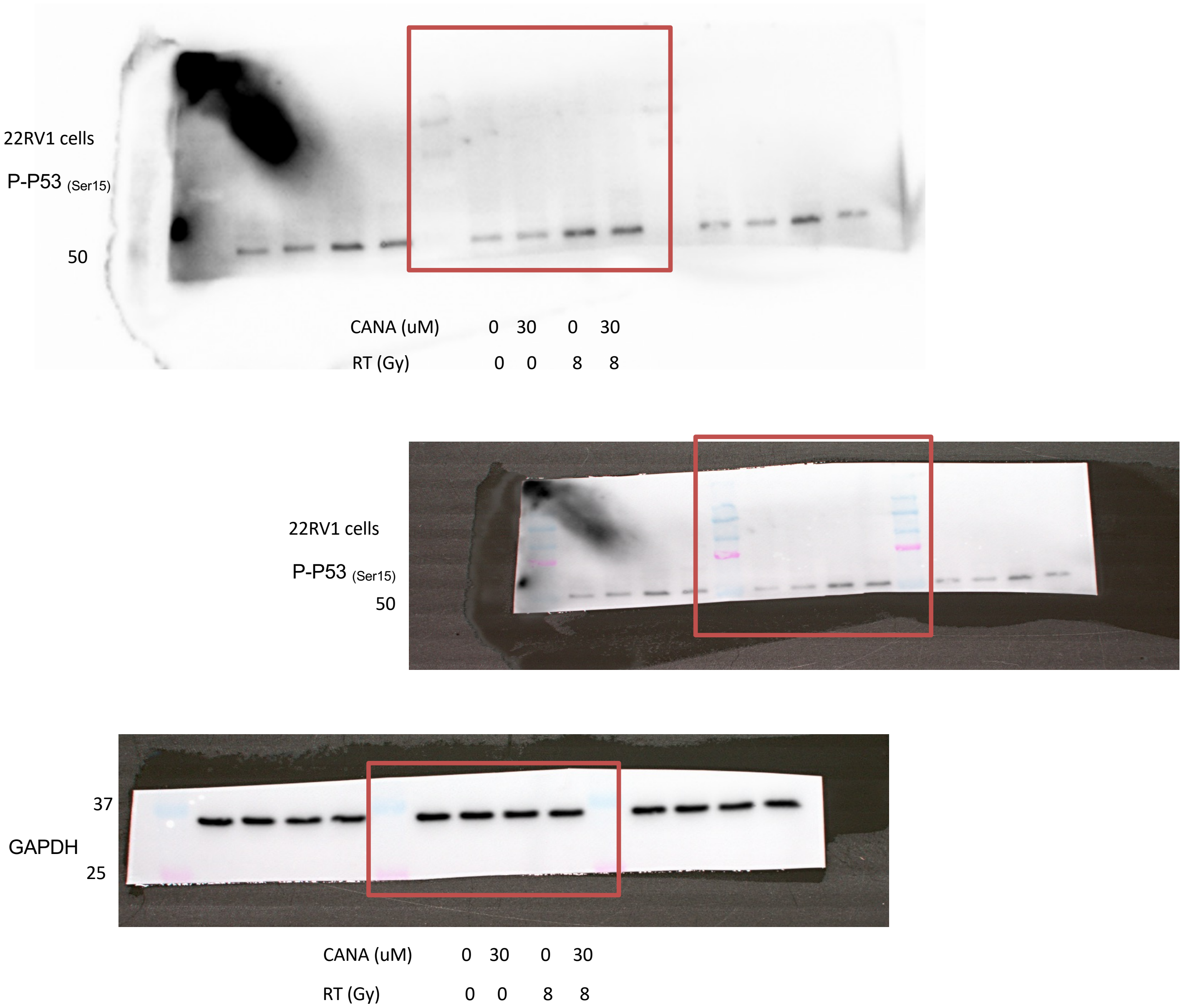

Supplementary Figure 6. Supplementary blots g: The supplementary blots 7 contain the original blots for Figure 7d for 22RV1 cells, including P21 and phosphorylated-P53 at ser15, and their representative GAPDH and b-actin control blots. The blots on the right side shows the visible molecular weight ladder. Figures may have a different aspect ratio than the image in the manuscript due to the size of the blot, as we are showing the full image.

Supplementary Tables

a

Cell line / (genes regulated)	Transcription factor (TF)	Normalized enrichment score (NES)	RNAseq Rank	Number of target genes
**PC3 CANA (Upregulated)**	ELF3	6.4	11.3	695
	MXI1	4.9	2.15	258
**PC3 CANA (Downregulated)**	ELK1	3.847	-2.67	405
	E2F2	4.4	-5.64	235
	GATA2	3.8	-3.45	235
	E2F3	4.4	-2.5	200
**22RV1 CANA (Upregulated)**	ATF3	3.362	1.61	12
**22RV1 CANA (Downregulated)**	MEF2D	5.277	-1.301	45
	PTPMT1	5.017	-1.61	19
**22RV1 (RT-CANA+RT) (Upregulated)**	ATF4	8.662	6.39	96
	ELF3	4.98	6.23	216
	FOS	4.7	4.11	46
	ATF3	7.639	2.7	134
**22RV1 (RT-CANA+RT) (Downregulated)**	E2F7	9.5	-6.87	241
	E2F1	9.5	-5.4	241
	E2F2	11.5	-4.1	246
	TFDP1	8.3	-3.3	117

Supplementary Table 1. Transcription factors. The table contains a list of significantly up- or down-regulated transcription factors identified in RNAseq data from non-irradiated PC3 cells and 22RV1 cells treated with (10μM) canagliflozin (CANA) (comparison: CANA vs control) and from irradiated (RT) (5Gy) 22RV1 cells (comparison: RT vs CANA+RT). All genes regulated significantly by the specified treatment (FDR q-value < 0.05) are selected from RNAseq data and entered into the iRegulon module (Cytoscape) to detect transcription factors associated with the specific gene expression pattern.

Antibodies used for Immunoblotting	
primary Antibody/ catalog no/ dilution	2° Antibody/ catalog no/ dilution
AMPKa #2532S 1:1000	Anti-Rabbit IgG, HRPlinked antibody #7074S 1:10000
P-AMPK (Thr172) #2532S 1:1000	
Raptor #2280S 1:1000	
P-RAPTOR (Ser792) #2083S 1:1000	
P-Akt (Ser473) #4058S 1:1000	
P-Akt (Thr308) #9275S 1:1000	
Akt #9272S 1:1000	
mTOR #2983S 1:1000	
P-mTOR (Ser2448) #2971S 1:1000	
p70S6K #9202S 1:1000	
P-P70S6K (Thr389) #9205S 1:1000	
S6 #2217S 1:1000	
P-S6 (Ser240/244) #2215S 1:1000	
4E-BP1 #9644S 1:1000	
P-4E-BP1 (Ser65) #9451S 1:1000	
HIF-1α #36169S 1:1000	
P27kip1 #3688S 1:1000	
p21cip1 #2947S 1:1000	
H3 #4499S 1:1000	
P-H3 (Ser10) #53348S 1:1000	
ERK #4695S 1:1000	
P-ERK (Thr202/Tyr204) #4370S 1:1000	
ACC #3662S 1:1000	
P-ACC (Ser79) #11818 1:1000	
P-P53 (Ser15) #9284 1:1000	
GAPDH (14C10) #5174S 1:5000	
B-actin (13E5) #5125S 1:1000	

Supplementary Table 2. Antibodies table. Primary and secondary antibodies used in immunoblotting assay. All antibodies were purchased from Cell Signaling Technology, Canada.
