# Supplementary material for: The SGLT2 inhibitor canagliflozin suppresses growth and enhances prostate cancer response to radiotherapy
Source: Commun Biol. 2023 Sep 8;6:919. doi: 10.1038/s42003-023-05289-w (PMC10491589; doi:10.1038/s42003-023-05289-w)
Supplement: Supplementary file 3 — Description of Additional Supplementary Files [file 42003_2023_5289_MOESM3_ESM.pdf]

Description of Additional Supplementary Files

File name: Supplementary data file 1
Description: PC3 cells treated with canagliflozin 10uM_RNAseq GSEA Pathway analysis.

File name: Supplementary data file 2
Description: Irradiated 22RV1 with 5Gy treated with canagliflozin 10uM_ RNAseq GSEA Pathway analysis.

File name: Supplementary data file 3
Description: PC3 cells treated with canagliflozin (10uM) for 24Hr_RNAseq_Feature count normalized numbers.

File name: Supplementary data file 4
Description: 22RV1 cells RNAseq_Feature count normalized numbers (24Hours post treatments) (RT (5Gy), Canagliflozin (10uM), and combination (RT+ Canagliflozin).

File name: Supplementary numerical source data
Description: Numerical source data for graphs/charts.
