# Supplementary material for: The SGLT2 inhibitor canagliflozin suppresses growth and enhances prostate cancer response to radiotherapy
Source: Commun Biol. 2023 Sep 8;6:919. doi: 10.1038/s42003-023-05289-w (PMC10491589; doi:10.1038/s42003-023-05289-w)
Supplement: Supplementary file 9 — Reporting Summary [file 42003_2023_5289_MOESM9_ESM.pdf]

nature portfolio

Corresponding author(s): Theos Tsakiridis

Last updated by author(s): Aug 2, 2023

Reporting Summary

Nature Portfolio wishes to improve the reproducibility of the work that we publish. This form provides structure for consistency and transparency in reporting. For further information on Nature Portfolio policies, see our Editorial Policies and the Editorial Policy Checklist.

Statistics

For all statistical analyses, confirm that the following items are present in the figure legend, table legend, main text, or Methods section.

n/a	Confirmed	
☐	☒	The exact sample size (*n*) for each experimental group/condition, given as a discrete number and unit of measurement
☐	☒	A statement on whether measurements were taken from distinct samples or whether the same sample was measured repeatedly
☐	☒	The statistical test(s) used AND whether they are one- or two-sided<br>*Only common tests should be described solely by name; describe more complex techniques in the Methods section.*
☐	☒	A description of all covariates tested
☐	☒	A description of any assumptions or corrections, such as tests of normality and adjustment for multiple comparisons
☐	☒	A full description of the statistical parameters including central tendency (e.g. means) or other basic estimates (e.g. regression coefficient) AND variation (e.g. standard deviation) or associated estimates of uncertainty (e.g. confidence intervals)
☐	☒	For null hypothesis testing, the test statistic (e.g. *F*, *t*, *r*) with confidence intervals, effect sizes, degrees of freedom and *P* value noted<br>*Give P values as exact values whenever suitable.*
☒	☐	For Bayesian analysis, information on the choice of priors and Markov chain Monte Carlo settings
☒	☐	For hierarchical and complex designs, identification of the appropriate level for tests and full reporting of outcomes
☒	☐	Estimates of effect sizes (e.g. Cohen's *d*, Pearson's *r*), indicating how they were calculated

Our web collection on statistics for biologists contains articles on many of the points above.

Software and code

Policy information about availability of computer code

Data collection

1- Agilent Technologies Seahorse XFe96 extracellular flux analyzer system (Santa Clara, CA), Wave software, version 2.6.1.56.
2- Next Gene Sequencing (NGS) (Illumina HiSeq 1500, Illumina, San Diego, CA) (Fancombe Institute, McMaster University): HiSeq 1500 Software version 2.2.68.
3- Cytoflex LX flow cytometer (Beckman Coulter, Mississauga, ON) (Core Flow Facility, McMaster University), CytExpert Software, version 2.4.
4- Vilber Fusion-FX imager (Marne-la-Vallée cedex 3, France), Fusion software version 18.02.
5- SpectraMax iD5 system (Molecular Devices, San Jose, California), SoftMax Pro 7 Software.
6- IHC images were acquired on an Olympus BX-40-F4 microscope (Breinigsville, PA) with Ach 40x/0.65 or Ach 10x/0.25 lens, using AmScope camera and acquisition software AmScope version 4.10.1.

Data analysis

1-Cell cycle data analysis was performed using FlowJo software (Version 10.8.0, FlowJo LLC, Ashland, OR).
2- Cell proliferation assay was performed at absorbance 762nm using SpectraMax iD5 system (Molecular Devices, San Jose, California) using SoftMax Pro 7 software.
3- Seahorse analysis: Agilent Seahorse Analytics data analysis application (web-base: https://seahorseanalytics.agilent.com/Account/Login) was used to analyse mitochondrial respiration assays and glycolytic rate assays for Agilent Seahorse XF Analyzer raw data output.
4- To determine the mode of interaction for therapy combinations (additivity, synergism, or antagonism) we used the SynergyFinder plus (https://synergyfinder.org) with the Highest Single Agent mathematical modelling (HSA) (open source).
5- RAD-ADAPT version 1.0 software was used for modelling clonogenic assay data.
6- Immunoblotting membrane imaging: Fusion software, version 18.02.
7- Immunoblotting quantification and IHC quantification for Necrosis and Cleaved Caspase-3: Image J 2 (version 1.53t).
8- RNAseq analysis: The use-galaxy platform (https://usegalaxy.org/) was used for raw sequencing data analysis.
9- Gene Set Enrichment Analysis: GSEA software Version 4.2.1.

10- Heat maps were generated using Morpheus software (open source web-base software at "https://software.broadinstitute.org/morpheus").
11- Transcription factor analysis: Cytoscape (version 3.9.1) plug in iRegulon (version 1.3).
12- The ProgGeneV2 (http://www.progtools.net/gene/index.php) and Prostate Cancer Transcriptome Atlas (PCTA) (http://www.thepcta.org) engines were used to analyze open-source mRNA expression and survival data from the Swedish-Watchful-Waiting cohort and GSE16560 vs The Cancer Genome Atlas (TCGA) and Cancer Research UK Cambridge Institute cohort (GSE70769), respectively.
13- All graphs/statistic analysis were generated/performed using GraphPad Prism software (Version 9, San Diego, CA).
14- Schematic graphs were created with BioRender.com under the agreement number: PN25FQG4H5 for figure 2a and the agreement number: PK25FRK4UN for figure 10.

For manuscripts utilizing custom algorithms or software that are central to the research but not yet described in published literature, software must be made available to editors and reviewers. We strongly encourage code deposition in a community repository (e.g. GitHub). See the Nature Portfolio guidelines for submitting code & software for further information.

Data

Policy information about availability of data

All manuscripts must include a data availability statement. This statement should provide the following information, where applicable:

- Accession codes, unique identifiers, or web links for publicly available datasets
- A description of any restrictions on data availability
- For clinical datasets or third party data, please ensure that the statement adheres to our policy

The RNA-seq data, including both raw data and normalized data, has been submitted to the Gene Expression Omnibus (GEO) under the accession number GSE:239688.

Human research participants

Policy information about studies involving human research participants and Sex and Gender in Research.

Reporting on sex and gender	N/A
Population characteristics	N/A
Recruitment	N/A
Ethics oversight	N/A

Note that full information on the approval of the study protocol must also be provided in the manuscript.

Field-specific reporting

Please select the one below that is the best fit for your research. If you are not sure, read the appropriate sections before making your selection.

☒ Life sciences ☐ Behavioural & social sciences ☐ Ecological, evolutionary & environmental sciences

For a reference copy of the document with all sections, see nature.com/documents/nr-reporting-summary-flat.pdf

Life sciences study design

All studies must disclose on these points even when the disclosure is negative.

Sample size	No formal sample size calculations were used to predetermine sample size in the animal experimental groups; Our in vitro experiments sample sizes are similar to those reported in our previous publication: Villani L.A. et al. The diabetes medication Canagliflozin reduces cancer cell proliferation by inhibiting mitochondrial complex-I supported respiration. Mol Metab. 2016 Oct; 5(10): 1048–1056. doi: 10.1016/j.molmet.2016.08.014.
Data exclusions	Data points were excluded if they were more than two standard deviations away from the mean, as this is the conventionally accepted method for identifying statistical outliers. This was predetermined exclusion criteria. outlier data calculated/determined with the Graphpad software calculator.
Replication	Each experiment's replicates are described in the figure legend. More information on each experiment is provided below:<br>-Proliferation assay (96 well plates): At least 3 different experiments (replicates) was performed, each included at least 6 duplicates,<br>-Clonogenic assay (12 well plates): At least 3 different experiments was performed, each included 3 duplicates,<br>-Seahorse analysis: 3 different experiments was performed.<br>-Cell cycle analysis: 3 different experiments was performed.<br>-Immunoblotting assay: At least 3 different experiments was performed.<br>-RNAseq analysis: 3 different replicates A,B,C for each treatment group.<br>-Animal study: 24 mice, 6 per group for Control, Canalization, Radiation, canalization + radiation.

	-IHC: For PHH3 quantification, investigators quantified 10 random HPFs for each tumor/slide and took average for it.<br>-For necrosis percentage evaluation, the whole tumor section was evaluated.
Randomization	Mice were randomized to treatment groups.
Blinding	Investigators were not blinded to the experiments (e.g., treatment groups). Blinding was not necessary as all measurements were objective. For animal experiments blinding was not possible as the investigators were administering treatments, but Investigators were blinded during histology processing and analysis.

Reporting for specific materials, systems and methods

We require information from authors about some types of materials, experimental systems and methods used in many studies. Here, indicate whether each material, system or method listed is relevant to your study. If you are not sure if a list item applies to your research, read the appropriate section before selecting a response.

Materials & experimental systems

n/a	Involved in the study
☐	☒ Antibodies
☐	☒ Eukaryotic cell lines
☒	☐ Palaeontology and archaeology
☐	☒ Animals and other organisms
☒	☐ Clinical data
☒	☐ Dual use research of concern

Methods

n/a	Involved in the study
☒	☐ ChIP-seq
☐	☒ Flow cytometry
☒	☐ MRI-based neuroimaging

Antibodies

Antibodies used

All antibodies were purchased from cell signaling company (New England BioLabs):
AMPKa cat #2532S, lot #21, dilution 1:1000, Polyclonal antibody
, Phospho-AMPK (Thr172) (40H9) cat #2532S, lot #27, dilution 1:1000, Monoclonal Antibody
, Phospho-RAPTOR (Ser792) cat #2083S, lot #7, dilution 1:1000, Polyclonal Antibody
, Raptor, cat#2280S, lot#13, dilution 1:1000, Monoclonal Antibody
, Phospho-Akt (Ser473), (193H12), cat #4058S, dilution 1:1000, Monoclonal Antibody
, Phospho-Akt (Thr308) cat #9275S, lot #27 dilution 1:1000, Polyclonal Antibody
, Akt cat #9272S, lot #30, dilution 1:1000, Polyclonal Antibody
, Phospho-mTOR (Ser2448), cat #2971S, lot #28, dilution 1:1000, Polyclonal Antibody
, mTOR, cat#2983s, lot#21, dilution 1:1000, Monoclonal Antibody
, p70 S6 Kinase, cat #9202S, lot#21, dilution 1:1000, Polyclonal Antibody
, Phospho-P70 S6 Kinase (Thr389), cat #9205S, lot#26, dilution 1:1000, Polyclonal Antibody
, S6 Ribosomal protein (5G10), cat #2217S, lot #10, dilution 1:1000, Monoclonal Antibody
, Phospho-S6 Ribosomal protein (Ser240/244), cat #2215S, lot #18, dilution 1:1000, Polyclonal Antibody
, 4E-BP1 (53H11), cat #9644S, lot #12, dilution 1:1000, Monoclonal Antibody
, Phospho-4E-BP1 (Ser65) cat #9451S, lot #12, dilution 1:1000, Polyclonal Antibody
, HIF-1α (D1S7W) XP, cat #36169S, lot #4, dilution 1:1000, Monoclonal Antibody
, P27 kip1 (D37H1), cat #3688S, dilution 1:1000, Monoclonal Antibody
, p21 waf1/cip1 (12D1), cat #2947S, lot #12, dilution 1:1000, Monoclonal Antibody
, Histone H3 (D1H2) XP, cat #4499S, lot #20, dilution 1:1000, Monoclonal Antibody
, Phospho-Histone H3 (Ser10) (D7N8E) cat #53348S, lot #1, dilution 1:1000, Monoclonal Antibody
, p44/42 MAPK (Erk1/2) (137F5), cat #4695S, lot #35, dilution 1:1000, Monoclonal Antibody
, Phospho-p44/42 MAPK (Erk1/2) (Thr202/Tyr204) (D13.14.4E) XP, cat #4370S, lot #28, dilution 1:1000, Monoclonal Antibody
, Acetyl-CoA Carboxylase, cat #3662S, lot #8, dilution 1:1000, Polyclonal Antibody
, Phospho-Acetyl-CoA Carboxylase (Ser79) (D7D11), cat #11818, lot #6, dilution 1:1000, Monoclonal Antibody
, Phospho-p53 (Ser15), cat #9284, lot#22, dilution 1:1000, Polyclonal Antibody
, GAPDH (D16H11) XP and (14C10), cat #5174S and #2118, lot #8 and #16, dilution 1:1000, Monoclonal Antibody
, B-actin HRP Conjugate (13E5), cat #5125S, dilution 1:1000, Monoclonal Antibody
, Anti-rabbit IgG, HRP-linked Antibody, cat #7074S, lot #31, dilution 1:10000, Secondary Antibody

Validation

All antibodies are commercially available and validation statements can be found on the manufacturer's website (Cell Signaling Technology, Inc.).

Eukaryotic cell lines

Policy information about cell lines and Sex and Gender in Research

Cell line source(s)

Human prostate cancer cell lines: PC3, 22RV1 and DU145 cells were purchased from ATCC.
-DU145-RR (radio-resistance) cells were generated by serially treating (DU145 cells, from ATCC) with 2Gy daily fractions of RT (Monday - Friday) to a total of 118Gy.
-human prostate cancer cell line: LnCap cells were provided by Dr. Damu Tang, (McMaster University, originally purchased from ATCC).

	PC3 cells were cultured in RPMI and 22RV1 and LnCap in ATCC-modified RPMI media supplemented with 10% FBS and 1% penicillin-streptomycin for LnCaps or 1% antibiotic-anti-mycotic for 22RV1 cells. DU145 and DU45-RR cells were cultured in DMEM media supplemented with 10% FBS and 1% antibiotic-anti-mycotic.
Authentication	The cell lines were authenticated (Cell Line Authentication Service, ATCC).<br>Cells were also authenticated using short tandem repeat DNA profiling, and the amplified DNA sequences were compared to the reference cell database, with a match of more than 80% being acceptable.
Mycoplasma contamination	All cells were tested negative for mycoplasma infection.
Commonly misidentified lines (See ICLAC register)	No commonly misidentified cell lines were used in the study.

Animals and other research organisms

Policy information about studies involving animals; ARRIVE guidelines recommended for reporting animal research, and Sex and Gender in Research

Laboratory animals	6–8 week old BALB/C nude mice and 6–8 week NOD-congenic (NRG) mice. All animals were housed under standard, regulated conditions following the guidelines of animal facility (McMaster University). Temperature range (20-25 degree c), Humidity range: 20-70%, 12 hour light cycle (on = 6am-6pm, off = 6pm - 6am).
Wild animals	Study did not involved wild animals.
Reporting on sex	Male (NRG and BLAB/C nude) mice are used because prostate cancer only affects male reproductive organs.
Field-collected samples	Following the guidelines of animal research (AUP # 16-12-41 and 20-12-47), animals were euthanized under anaesthesia by cervical dislocation at the end of the experiment. Following sacrifice, predetermined organs, fluids, and/or tissues were harvested, rinsed, placed in pre-weighed counting tubes, and weighed. Blood and urine samples were collected at the end point. The extracted tissues/ organs were stored in -80 C or formaldehyde.
Ethics oversight	All experiments were approved by the McMaster Animal Ethics Committee and conducted following the guidelines of animal research (AUP # 16-12-41 and 20-12-47).

Note that full information on the approval of the study protocol must also be provided in the manuscript.

Flow Cytometry

Plots

Confirm that:

☐ The axis labels state the marker and fluorochrome used (e.g. CD4-FITC).

☐ The axis scales are clearly visible. Include numbers along axes only for bottom left plot of group (a 'group' is an analysis of identical markers).

☐ All plots are contour plots with outliers or pseudocolor plots.

☒ A numerical value for number of cells or percentage (with statistics) is provided.

Methodology

Sample preparation	Human cell lines: PC3 and 22RV1 cells. Both cells were seeded in a 10cm dishes (0.5x106–2x106/dish), incubated overnight and treated without or with canagliflozin (CANA) (0-30µM) and or radiotherapy (RT) (0-8Gy). Then cells were incubated for 48 hours to a 50-60% confluent. Then cells were harvested and washed with cold PBS buffer, fixed in 70% ethanol, and stored at −20°C. Before analysis, cells were centrifuged, ethanol was aspirated, cells were washed with PBS and stained with propidium-iodide (ThermoFisher FxCycle PI) used to stain cells for 30 minutes. Data collection. Samples were analyzed by flow cytometry using CytExpert software version 2.4. at least 10.000 events were acquired per sample.
Instrument	Cytoflex LX flow cytometer (Beckman Coulter, Mississauga, ON) (Core Flow Facility, McMaster University).
Software	Data collection : CytExpert software version 2.4.<br>Data analysis was performed using FlowJo software (Version 10.8.0, FlowJo LLC, Ashland, OR).
Cell population abundance	No sorting was performed with the flow cytometer.
Gating strategy	We used forward scatter and side scatter to find viable, single cell events. This cell population gate was placed on PI-area vs PI-height where we eliminated doublets from the analysis.

☐ Tick this box to confirm that a figure exemplifying the gating strategy is provided in the Supplementary Information.
